# Relationship between Morbidity and Health Behavior in Chronic Diseases

**DOI:** 10.3390/jcm9010121

**Published:** 2020-01-02

**Authors:** Munjae Lee, Sewon Park, Kyu-Sung Lee

**Affiliations:** 1 Research Institute for Future Medicine, Samsung Medical Center, Seoul 06351, Korea; emunjae@skku.edu (M.L.); se10919@g.skku.edu (S.P.); 2 Department of Medical Device Management and Research, SAIHST, Sungkyunkwan University, Seoul 06355, Korea; 3 Department of Urology, Samsung Medical Center, Sungkyunkwan University School of Medicine, Seoul 06351, Korea

**Keywords:** chronic disease, health behavior, socioeconomic status, primary care, Korea

## Abstract

This study aimed to analyze the demographic characteristics and health behaviors related to chronic diseases and to identify factors that may affect chronic diseases. Data from the Seventh Korea National Health and Nutrition Examination Survey were used, and 3795 adults aged above 40 years were included. The following demographic variables were obtained: sex, age, education, income, type of health insurance, and private insurance. The following health behavior factors were also analyzed: medical checkup, drinking, smoking, exercise, obesity, and hypercholesterolemia. Participants with lower socioeconomic status had a higher risk of developing chronic diseases. Meanwhile, those with private health insurance had a lower risk of developing chronic diseases. In addition, participants who underwent medical checkups and performed exercises had a lower risk, while those with obesity and hypercholesterolemia had a higher risk of developing chronic diseases. It is necessary to manage chronic diseases through comprehensive programs, rather than managing these diseases individually, and through community primary care institutions to improve health behaviors.

## 1. Introduction

An individual’s behavior related to health may have an effect on their physical health or ability to recover from illness. In particular, health-related behavior, such as a lack of exercise, smoking, and drinking, are some of the main factors that can contribute to morbidity and mortality [1,2,3]. Health behavior affects 40% of premature deaths; in order to reduce premature mortality, improving health behaviors is more cost-effective than improving the social and physical environments or health-care systems [4]. These health behaviors are important in maintaining good health, which is influenced by biological and socioeconomic factors, among others [5,6]. Rapid economic growth, high health-care costs, lifestyle changes, and population aging have been associated with an increased prevalence of chronic diseases worldwide. Chronic diseases may cause complications, and thus, require continuous care and are among the types of diseases with high health-care costs due to their long disease duration [7,8,9]. 

Chronic diseases, one of the leading causes of death worldwide, especially cardiocerebrovascular diseases, diabetes, and hypertension, have a high mortality rate. However, the mortality rate of chronic diseases can be reduced through prevention [10,11,12]. Chronic disease is closely related to changes in health behaviors; the main health behaviors affecting the development of chronic diseases include health risk behavioral factors, such as smoking, drinking, and physical activities, and clinicopathologic factors, including obesity, hypertension, and hypercholesterolemia [13,14]. In particular, since health-related lifestyles have increased the risk of mortality, the significance of managing health risk behavioral factors has also been increasing. Thus, it is necessary to prevent chronic diseases and delay the aggravation of symptoms by improving individual lifestyles [15,16,17]. In addition, individual health behaviors may differ according to sociodemographic characteristics including age and sex [18]. In the identification of the individual physical condition, sociodemographic and socioeconomic factors are known to act as important factors, and prevalence rates vary in accordance with the individual’s income level, education level, and socioeconomic factors [19]. 

Previous studies have analyzed the relationship between chronic diseases and health promotion behaviors, but were only conducted in predetermined age groups, such as in older patients, or examined the relationship between chronic diseases and health behaviors while only targeting certain chronic diseases [20,21,22,23,24]. As the number of polychronic patients has increased, a comprehensive analysis of chronic diseases is required. To date, the number of studies evaluating patients with chronic diseases is limited. Accordingly, in this study, we aimed to analyze the sociodemographic characteristics and health behaviors related to the development of chronic diseases and to identify factors that may have an effect on the morbidity of chronic diseases. Through this and by suggesting measures to contribute to the effective management and prevention of chronic diseases, we intend to promote the health of the people. 

## 2. Experimental Section

### 2.1. Data Source and Research Participants

**Figure 1:**
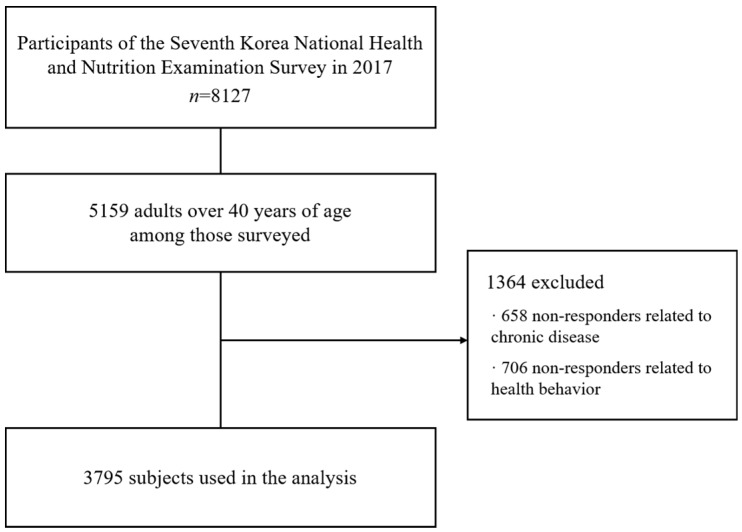
Flowchart of the study.

In this study, we utilized the source data from the 2nd year (2017) of the 7th period of the Korea National Health and Nutrition Examination Survey, performed by the Korea Centers for Disease Control and Prevention. The Korea National Health and Nutrition Examination Survey (KNHANES) is a nationwide national survey, conducted to determine health-related parameters including the prevalence of chronic diseases and health behaviors based on Article 16 of the National Health Promotion Act. A total of 10,430 individuals from 3580 households were surveyed, but only 8127 participated in the study. Of them, 5159 aged above 40 years were extracted. In addition, 658 individuals whose answers were not related to chronic diseases were excluded; hence, only 3795 participants were analyzed, after further excluding 706 who did not respond to the questions related to health behaviors. The KNHANES is approved by the ethical committee of the Korea Centers for Disease Control and Prevention. The requirement for informed consent was waived because data in the KNHANE database are anonymized in adherence to strict confidentiality guidelines. The flowchart is shown in Figure 1.

### 2.2. Description of Variables

In this study, questions related to sociodemographic characteristics, morbidity, and presence of chronic diseases, and health behaviors were utilized. Sex, age, education level, level of income, type of health insurance, and private insurance policy were used as sociodemographic variables. In terms of age, participants aged 19 years or older were divided into two groups: adults and older adults (aged 65 years and above). The education level was stratified into middle school graduates or less and high school graduates or more. Income status was determined by the monthly mean household gross income and was classified based on 3 million won as the cut-off point. The patients with health insurance were classified as health insurance subscribers and medical care beneficiaries [23,25,26,27,28]. 

Hypertension and diabetes are the main causes of cardiovascular disease, and the number of patients continues to rise due to the increase in obesity rate. In addition, the cost of medical care is proliferating more rapidly than the number of patients. Therefore, it is significant to prevent it by analyzing the factors influencing chronic diseases. Hitherto, chronic disease was defined as hypertension, dyslipidemia, stroke, myocardial infarction, and diabetes. The presence of chronic disease was determined based on a response of “Yes” to the question related to a doctor’s diagnosis. Health behaviors included health checkups, drinking, smoking, exercise, obesity, and hypercholesterolemia [28,29,30,31]. Health checkup status was classified as patients who underwent health checkups and those who did not undergo health checkups. Drinking status was classified as non-drinkers and drinkers based on the monthly drinking rate; smoking status was classified as non-smokers and smokers using the current smoking rate. Exercise history was stratified as those who performed exercises and those who did not perform exercises based on the aerobic physical activity practice rate [32,33]. Furthermore, the prevalence of obesity was determined and obesity was stratified based on the following indices: a body mass index of 18.5 kg/m^2^ or higher or a body mass index of 23 kg/m^2^ or lower indicates normal weight, while a body mass index of 25 kg/m^2^ or higher indicates obesity [34,35,36]. Hypercholesterolemia was stratified based on its prevalence (Table 1).

### 2.3. Statistical Analysis 

In order to analyze the relationship among sociodemographic characteristics, health behaviors, and the presence of chronic diseases, statistical analyses were conducted using the SPSS (version 25.0, https://www.ibm.com/kr-ko/analytics/spss-statistics-software). 

First, cross-analysis was performed to analyze the relationship between chronic diseases and sociodemographic characteristics and between health behaviors and chronic diseases. In order to determine the relationship between sociodemographic characteristics and health behaviors and the risk for developing chronic diseases, a logistic regression analysis was performed.

## 3. Results

### 3.1. Participants’ Demographic Characteristics

Of the total participants, 56% were women and the proportion of women was higher than that of men; older adults aged 65 years or higher accounted for 33% of the total study population. Most of the participants were middle school graduates or had obtained higher education (2269 persons, 59.8%) and had an income of more than 3 million won (2107 persons, 55.5%). With regard to the type of health insurance, national health insurance subscribers accounted for 95.7% of the total participants according to the characteristics of health insurance in Korea. Meanwhile, private insurance subscribers accounted for 74.2%, even though the proportion of health insurance subscribers corresponded to a majority; this finding indicates that most of the patients took a private medical insurance policy due to the lack of coverage by the national health insurance. A total of 857 patients (22.6%) underwent medical checkups, which suggests that only a few patients were able to undergo medical checkups. A total of 1762 patients (46.4%) developed chronic diseases, of whom 21.4% had two or more chronic diseases (Table 2).

### 3.2. Relationship between Demographic Characteristics and Chronic Diseases

In this study, we intended to analyze the relationship between sociodemographic characteristics and chronic diseases, and the results are shown in Table 3. Among chronic disease patients, 955 (44.9%) were women, this proportion being higher than that of men. Meanwhile, 807 (48.4%) of 862 male participants had chronic diseases, which indicates that men had a higher rate of chronic disease morbidity. Approximately 71.3% of the participants aged 65 years or higher had chronic diseases. In addition, most of the patients with a lower educational level and lower-income level had chronic diseases (981 patients (64.3%)and 1008 patients (59.7%), respectively). A total of 1648 (45.4%) health insurance subscribers were chronic disease patients, while 114 (69.9%) medical care recipients were chronic disease patients. Furthermore, 1128 (40.1%) chronic disease patients were private insurance subscribers.

### 3.3. Relationship between Health Behavior and Chronic Diseases 

We analyzed the relationship between health behaviors and chronic diseases; the results are shown in Table 4. Of the total chronic disease patients, 944 (50.6%) were alcohol drinkers, 1503 (47%) were smokers, and 605 (40.1%) performed exercises, which is less than the number of patients who did not perform exercises (1157, 50.6%). Moreover, 1263 (52.7%) and 823 (73.9%) patients with obesity and hypercholesterolemia, respectively, had chronic diseases. 

### 3.4. Factors Affecting Chronic Diseases

In order to determine the factors that may affect the development of chronic diseases, logistic regression analysis was performed, and the results are shown in Table 5. The factors with statistically significant effects in patients with chronic disease included sex, age, education, income, types of health insurance, decision to take a private insurance policy, health checkups, exercise, obesity, and hypercholesterolemia. 

In men, the risk of developing chronic diseases was higher by 1.498 times. Further, as age increased, the risk of developing chronic diseases also increased by 3.145 times. In participants with a higher education level, the risk of developing chronic diseases increased by 0.535 times. In participants with higher income, the risk of developing chronic disease reduced by 0.773 times. With regard to the type of health insurance, the risk of developing chronic diseases increased by 1.727 times among medical care beneficiaries. In addition, for those who took a private insurance policy, the risk of developing chronic diseases increased by 0.782 times. Meanwhile, the risk of developing chronic diseases decreased by 0.782 times and 0.861 times among those who underwent medical checkups and who performed exercises, respectively. In normal-weight people, the risk of developing chronic diseases reduced by 0.544 times. In patients with hypercholesterolemia, the risk increased by 5.444 times.

## 4. Discussion

In this study, we analyzed the factors affecting the development of chronic diseases through logistic regression analysis using the data from the Korea National Health and Nutrition Examination Survey (2017). Of the sociodemographic characteristics, sex, age, education and income level, types of health insurance, and private insurance were found to have an effect on chronic diseases. In terms of sex, the proportion of women with chronic diseases was higher than that of men. Compared with women, men had a higher rate of chronic disease morbidity and the risk of developing chronic diseases. These results are inconsistent with those of previous studies, which reported that the prevalence of chronic diseases is higher among women than in men because men can maintain their economic level for longer than women. Women who have a lower income level than men have relatively low medical accessibility and find it difficult to manage their chronic diseases [37]. The number of chronic disease patients is increasing due to the lack of physical activity and the increasing prevalence of hypercholesterolemia and obesity, and considering that previous studies have shown that the prevalence of chronic disease was lower among men who received management, managing chronic diseases according to sex seems to be of utmost importance [38]. In addition, the number of patients aged 65 years or older who had chronic diseases was higher; therefore, the higher the age, the higher the risk of developing chronic diseases. This finding is consistent with those of a previous study, which reported that as age increases, the prevalence of chronic diseases also increases due to the decreased amount of physical activities and habit-based health risk behaviors [8,39]. 

It was also found that the higher the income and education levels, the lower the risk of chronic diseases. This finding is consistent with those of previous studies reporting that socioeconomic status, including income, education, and occupation levels, affects the health-related lifestyles and risk of chronic diseases [40]. Because of the low rates of physical activity and exercise practice and as the provision of medical services for managing chronic diseases has still not been ensured owing to lower educational levels or living standards, the prevalence of chronic diseases is increasing. Among medical care beneficiaries, the risk of developing chronic diseases was high, which was similar to the results of a previous study reporting that the incidence of chronic disease increased among individuals who belonged to the lower social class, like those in the low-income bracket. Social determinants, such as income, education, and social class, may cause health-related inequality but create an environment in which quality medical care can be provided for the treatment of chronic diseases. In addition, non-medical factors, such as social determinants, play a more substantial role in the management of chronic diseases than medical factors. It seems that medical care beneficiaries with low income may have more difficulty in managing chronic diseases [41,42,43]. There were many chronic disease patients who obtained a private medical insurance policy; the results showed that patients with private medical insurance had a lower risk of developing chronic diseases. These findings are similar to those of a previous study, which indicated that those who have private medical insurance policies tend to receive outpatient and inpatient treatments. In line with these findings, among patients with chronic diseases who require continuous health care, those with private medical insurance have a reduced burden in terms of medical expenses, leading to better health-care outcomes [44,45,46]. Considering these results, there are limitations in managing chronic diseases with national health insurance only. Furthermore, it is estimated that people obtain commercial medical insurance policies due to the burden of medical expenses caused by the recent increase in polychronic diseases. Therefore, since health-related inequalities in the low-income group patients, who find it difficult to pay the private medical insurance premiums, will become a serious problem if we only rely on private medical insurance for the management of chronic diseases, the coverage of the national health insurance should be reinforced for the management of chronic diseases. 

Among health behaviors, the factors affecting the risk of developing chronic diseases included health checkups, exercise, obesity, and hypercholesterolemia. Those who underwent periodic health checkups had a risk of developing chronic diseases, which is similar to previous findings showing that periodic health checkups promote health and help prevent chronic diseases [8,47]. In addition, considering the results of previous studies reporting that those who benefit from health insurance are more likely to receive health checkups depending on the nature of the health insurance system in Korea, chronic diseases could be effectively managed through modifying the nature of the insurance provided. Previous studies have shown that health behavior factors related to chronic diseases include smoking, drinking, exercise, body mass index, and regular life and eating habits [7,29,36,48,49]. However, in this study, drinking and smoking did not have a statistically significant effect on the prevalence of chronic diseases, and these results are different from those of existing research. Furthermore, exercise, obesity, and hypercholesterolemia were associated with the risk of developing chronic diseases, consistent with existing research. Among those who performed exercises, the risk of developing chronic diseases was lower, while among those with obesity and hypercholesterolemia, the risk of developing chronic diseases was higher. Weight loss via exercise programs reduces the risk of developing chronic diseases. Maintaining a standard body weight can prevent chronic diseases by alleviating hypercholesterolemia. Management of chronic diseases should be comprehensively performed with weight management through exercise; however, there seems to be a limitation in this regard according to patients’ behavioral changes [50,51]. In order to overcome this limitation, wearable medical devices, which use ICT (Information & Communication Technology), have recently been developed for chronic disease management. Prevention and management of chronic diseases can be ensured through exercise [52,53,54,55]. The use of medical devices to promote physical activity leads to obesity and hypercholesterolemia management, and through the linkage between these medical devices and local clinic-centered, effective management of chronic diseases can be achieved through periodic monitoring. The results of this study also suggest that gender, age, education, and income levels have impacts on chronic disease, and it is significant to add these as risk factors and to continue monitoring in local clinic-centered facilities. Through this, a personalized chronic disease management system could be established.

This study has some limitations. First, chronic disease patients aged 40 years or below were not included. Recently, the number of younger chronic disease patients has increased owing to changes in lifestyle, therefore, further studies to analyze the factors influencing the risk of developing chronic diseases in this age group will be required, with the patients stratified as follows: youth, middle-aged, and older adults. Second, analyses according to the number of chronic diseases were not performed. In this study, only the presence or absence of chronic diseases in patients was assessed. Further studies to determine the influencing factors according to the number of chronic diseases are required. Third, there was no analysis of factors affecting chronic disease according to the residential area. Accessibility to medical services varies depending on where you live; therefore, chronic disease management may be different. Hence, it is necessary to analyze the factors affecting chronic diseases according to urban and rural areas. Despite these limitations, we comprehensively analyzed the factors influencing the prevalence of chronic diseases. Our study is significant as we were able to determine the risk factors for chronic diseases, which can be used as a basis for developing policies for the comprehensive management of chronic diseases, based on sex, age, and social factors. 

## 5. Conclusions

In order to manage chronic diseases, the management approach should be based on patients’ socioeconomic characteristics to address the differences related to sex, education, income, and medical care. The management should also include approaches to improve health behaviors, including the use of wearable medical devices and digital healthcare products. Based on our findings, we presume that chronic diseases develop due to a combination of factors. Age, socioeconomic factors, obesity, and hypercholesterolemia are factors that can be controlled to prevent and manage chronic diseases through comprehensive programs rather than through individual management. Moreover, those who belong to the lower social class, are more likely to require chronic disease management via primary healthcare institutions in the community. In order to improve health behaviors, continuous observation is required, and local clinic-centered chronic disease management can help improve health behaviors. It is significant to establish a comprehensive management system and promote efficient medical delivery systems for chronic diseases focused on local clinic-centered facilities. However, Korea’s medical delivery system urgently needs reorganization due to the concentration of university hospitals and the weakening of a local clinic-centered structure. Therefore, in order to expand the role of local clinic-centered facilities and to efficiently manage chronic diseases, the integrated local clinic-centered care chronic disease management project is being implemented. Through this, medical treatment for chronic disease management and education for improving lifestyle, are applied to lower the patient’s copayment. If the burden reduction of chronic disease management is expanded, the dependency on private health insurance will be reduced, which will prevent excessive medical expenses for chronic patients. In addition, strengthening the role of local clinic-centered facilities will lead to strengthening medical access for low-income people, thereby relieving health inequalities. For older adults, when included in the community care project in line with community-based primary healthcare service, comprehensive management of chronic diseases, including health improvement and lifestyle modification, could be implemented. In particular, in Europe, where public health policies are in place, chronic diseases are effectively managed by strengthening the local clinic-centered services, such as the attending physician, to manage chronic diseases. For common goals such as chronic disease management, community care is implemented to ensure continuous health care. In view of this, chronic disease management through public health policy should be implemented prior to private medical insurance. Patients with private medical insurance have a lower risk of developing chronic diseases, but this can be seen as a problem of low insurance coverage for chronic diseases. This can be resolved through community care projects such as in Europe. Because of this, patient-centered chronic disease management will ultimately improve the health of chronic disease patients. 

## Figures and Tables

**Table 1:** Classification and definition of variables.

Variable	Definition
Sex	0 = Female
	1 = Male
Age	0 = Adult
	1 = Senior
Education	0 = ≤Middle school
	1 = ≥Middle school
Income	0 = <300
	1 = ≥300
Type of insurance	0 = National Health Insurance
	1 = Assistance
Private insurance	0 = No
	1 = Yes
Chronic disease	0 = No
	1 = Yes
Medical checkup	0 = No
	1 = Yes
Drinking	0 = Non-drinker
	1 = Drinker
Smoking	0 = Non-smoker
	1 = Smoker
Exercise	0 = No
	1 = Yes
Obesity	0 = Obesity
	1 = Normal
Hypercholesterolemia	0 = No
	1 = Yes

**Table 2:** Demographic characteristics (*n* = 3795).

Characteristic	Type	N	%
Sex	Female	2126	56.0
	Male	1669	44.0
Age	40–65	2544	67.0
	≥65	1251	33.0
Education	<Middle school	1526	40.2
	≥Middle school	2269	59.8
Income	<300	1688	44.5
	≥300	2107	55.5
Type of insurance	National health insurance	3632	95.7
	Assistance	163	4.3
Private insurance	Y	2814	74.2
	N	981	25.8
Medical checkup	Y	857	22.6
	N	2938	77.4
Chronic disease	0	2033	53.6
	1	950	25.0
	<2	812	21.4

**Table 3:** Relationship between demographic characteristics and chronic diseases.

Characteristic	Type	Chronic Disease				*p*-Value
		N	%	Y	%	
Sex **	Female	1171	55.1	955	44.9	0.019
	Male	862	51.6	807	48.4	
Age ***	40–65	1674	65.8	870	34.2	0.001
	≥65	359	28.7	892	71.3	
Education ***	<Middle school	545	35.7	981	64.3	0.001
	≥Middle school	1488	65.6	781	34.4	
Income ***	<300	680	40.3	1008	59.7	0.001
	≥300	1353	64.2	754	35.8	
Type of insurance ***	NHI	1984	54.6	1648	45.4	0.001
	Assistance	49	30.1	114	69.9	
Private insurance ***	Y	1686	59.9	1128	40.1	0.001
	N	347	35.4	634	64.6	

** *p* < 0.05, *** *p* < 0.001.

**Table 4:** Relationship between health behavior and chronic diseases.

Characteristic	Type	Chronic Disease				*p*-Value
		N	%	Y	%	
Medical checkup	Y	453	52.9	404	47.1	0.331
	N	1580	53.8	1358	46.2	
Drinking ***	Y	923	49.4	944	50.6	0.001
	N	1110	57.6	818	42.4	
Smoking *	Y	1696	53.0	1503	47.0	0.062
	N	337	56.5	259	43.5	
Exercise ***	Y	904	59.9	605	40.1	0.001
	N	1129	49.4	1157	50.6	
Obesity ***	Y	1132	47.3	1263	52.7	0.001
	N	901	64.4	499	35.6	
Hypercholesterolemia ***	Y	291	26.1	823	73.9	0.001
	N	1742	65.0	939	35.0	

* *p* < 0.1, *** *p* < 0.001.

**Table 5:** Factors affecting the development of chronic diseases.

Dependent Variable	Independent Variable	Exp(B)	*p*-Value
Chronic disease	Sex ***	1.498	0.001
	Age ***	3.145	0.001
	Education ***	0.535	0.001
	Income **	0.773	0.004
	Type of insurance **	1.727	0.008
	Private insurance **	0.803	0.036
	Medical checkup **	0.782	0.009
	Drinking	1.101	0.252
	Smoking	1.061	0.606
	Exercise *	0.861	0.060
	Obesity ***	0.544	0.001
	Hypercholesterolemia ***	5.444	0.001

* *p* < 0.1, ** *p* < 0.05, *** *p*< 0.001.
